# Grandmothers: Central scaffolding sources impacting maternal and infant feeding practices in Colombia

**DOI:** 10.1111/mcn.13162

**Published:** 2021-07-09

**Authors:** Natalia Concha, Sandra Jovchelovitch

**Affiliations:** ^1^ Department of Psychological and Behavioural Science The London School of Economics and Political Science London UK

**Keywords:** cultural context, family influences, infant feeding, poverty, qualitative methods

## Abstract

A growing body of evidence highlights that maternal and child nutrition programmes need to extend beyond the mother–child dyad by adopting a family systems approach, particularly in the Global South. Guided by a sociocultural and community psychology understanding of health, the paper explores factors identifying grandmothers as central resources for nutrition programmes. The study was conducted in a Colombian urban periphery applying a qualitative longitudinal design (prenatal and postpartum). It is based on interviews with adolescent mothers and mothers in their 20s (*n* = 35 at T1; *n* = 21 at T2), grandmothers (*n* = 15 at T1; *n* = 12 at T2) and community/public stakeholders (*n* = 17). Many of the participants live in low‐income households headed by grandmothers, who adjust feeding practices to the extent of their economic capacity. Findings reveal grandmothers play a central role in decision‐making and in enabling a holistic support system for the dyad. This is defined as grandmothers' scaffolding; it covers nutrition advice, breastfeeding and infant feeding, cultural practices, caregiving and maternal mental health. The study helps build the evidence‐base for the transferability of a family systems approach to Global South regions by using sociocultural and community psychology concepts to fortify the rationale for including grandmothers in maternal and child nutrition programmes. It argues for the need to continue raising the visibility of key actors like grandmothers and for nutrition programmes to align themselves more flexibly with the needs of families experiencing poverty.

**Key messages**

- The study contributes to current understandings of the grandmothers' support system as *scaffolding* the maternal–child dyad, in the form of nutrition advice, infant feeding practices, as well as caregiving and maternal mental health.
- Some grandmothers feed infants complementary foods to enable mothers to seek, or return to, work.
- Policy makers could consider greater flexibility when working with families in impoverished settings, by adapting programmes to better fit people's social realities and by supporting *the carers of caregivers*.

## 1 INTRODUCTION

Malnutrition and its related forms remain a global challenge. In the under‐five population, around 149 million children are stunted, and 49 million are underweight for their height (UNICEF, WHO, & WBG, 2019). Although South America has made progress compared with sub‐Saharan Africa and Asian regions, it continues to experience malnutrition. Despite regional efforts, in countries like Colombia, targets have not always been attained. The national prevalence of stunting for children under five is 12.6%, and 36.1% of infants less than 6 months old are exclusively breastfed, compared with the regional average of 57.3% (Micha et al., 2020). Addressing these challenges in unequal societies like Colombia (where this study takes place) is complex, owing to multiple factors (Bhutta et al., 2013; Black et al., 2013; Locks et al., 2015). At a macro level, such factors largely relate to socio‐economic conditions shaping health. At the micro level, they can be better understood when considering the family network rather than solely focusing on the maternal–child dyad. For the latter, family research has demonstrated how families are ‘practiced’ beyond the heteronormative model, which refers to the heterosexual dual parent nuclear structure (Morgan, 2011). A family systems approach regards the maternal–child dyad within a wider relational, cultural and interdependent system, with other members who influence health knowledge and practices (Kitzman‐Ulrich et al., 2010; Novilla et al., 2020). Applying a family systems approach to public health recognises key social actors, who impact health enhancing behaviours (Novilla et al., 2020). In the maternal and child nutrition (MCN) literature, Aubel (2012) has identified the need to go beyond the dyad and open‐up programmes to include the family and community. She has highlighted the central role grandmothers have as maternal advisors and active caregivers (Aubel, 2012; Aubel et al., 2004). A few studies have focused on the negative influences of grandmothers on MCN, particularly in exclusive breastfeeding cessation (Pilkauskas, 2014; Susin et al., 2005). Yet global health research has found that grandmothers positively influence breastfeeding and complementary feeding practices (Bezner Kerr et al., 2008; Faye et al., 2019; Gupta et al., 2015; Ijumba et al., 2014; Karmacharya et al., 2017; MacDonald et al., 2019; Martin et al., 2015). Grandmothers' influence and support is relevant given the limited involvement fathers tend to have in MCN in Global South populations (Aubel, 2012; MacDonald et al., 2019; World Health Organization, 2015). Yet, apart from some MCN grandmother initiatives adopting family‐centred approaches, wider uptake is still needed (Aubel et al., 2004; Faye et al., 2019; MacDonald et al., 2019). In Colombia, families are often mono‐parental, making the role of grandmothers essential to support the dyad (Cháves, 2019).

This paper contributes to the family systems approach adopted in the MCN literature with a qualitative study in peripheral ‘barrios’ (neighbourhoods) in Colombia. It argues that including grandmothers in MCN programmes is relevant in these areas, as many families differ from the heteronormative model. Focusing on key actors like grandmothers has potential given the support they have been found to provide in MCN and health (Aubel, 2012; Aubel et al., 2004; MacDonald et al., 2019).

### 1.1 A sociocultural and community health psychology approach

A family systems approach may be more appropriate in addressing MCN in settings where different family members are responsible and involved in caregiving. Yet MCN programmes have traditionally targeted the dyad, and this has been attributed to an individualistic focus in the public health field (Aubel, 2012). Such a focus contrasts with the broader individualisation critique at the forefront of community health psychology (CHP), which aims to expose inequalities by adopting a relational, cultural, contextual and structural view of health (Campbell & Cornish, 2012, 2014; Murray & Poland, 2006). CHP calls for greater recognition of the central role communities, marginalised groups and other stakeholders can have in addressing such inequalities (Campbell & Cornish, 2014; Nolas, 2014). CHP traditionally has not embraced complexity by relying on the simplicity of the onion model, which illustrates how people are situated in society by forming distinct layers from the individual to the structural (Campbell & Cornish, 2014). More recently, the discipline has called on scholars to recognise the ‘messiness’ (Nolas, 2014) inherent in the interrelated factors impacting health. This equates with poverty added to the social determinants of health model [Commission on Social Determinants of Health (CSDH), 2008; Marmot, 2005] along with psychological and cultural dynamics shaping health practices (Campbell, 2014, 2020). Additionally, Lund et al. (2018) have called for more qualitative evidence to understand how context and culture can better inform local mental health initiatives in impoverished settings. Maternal mental health has been identified as a risk factor impacting MCN in low‐income households (Black et al., 2013), given stressors related to socio‐economic disadvantage (Herba et al., 2016; Rahman et al., 2008; Ramakrishnan, 2011; WHO, UN Children's Fund, & WBG, 2018). Apart from unaffordability of nutritional foods, maternal mental health—particularly depression—may affect caregiving, including feeding practices (Anato et al., 2019; Herba et al., 2016). This paper argues that efforts to address MCN should consider a more holistic understanding of maternal caregivers. This involves recognising the socio‐economic circumstances which may be limiting MCN practices on the one hand, and counteracting them with the supportive responses available for mothers and children on the other.

In addition to CHP, a family systems approach to MCN can be further informed by a sociocultural psychology concept termed ‘psychosocial scaffoldings’ (Jovchelovitch & Priego‐Hernández, 2013). Psychosocial scaffoldings describe the actions and mechanisms of support which can help youth confront poverty and adversity. The concept comes from research identifying the role of family and community actors in protecting youth in Brazilian favelas (Jovchelovitch & Priego‐Hernández, 2013). It originates from Vygotsky's (1980, 1986) and Bruner's (1978, 1983, 1986) social development theories, which illustrate how an experienced adult or peer can build pathways to support the developmental trajectory of the child. Applied to a family systems approach, it serves as a useful tool by extending chains of support from mother–infant to identify who is supporting the mother. It is relevant to maternal research in South America showing grandmothers' support (Carvalho et al., 2008; Ferreira Takei et al., 2012; Oliveira & Bastos, 2000; Tenorio, 2012), in contexts where many households are intergenerational (i.e., three to four generations under one roof) and headed by women (Chant & Craske, 2003; Climent, 2012; Cuesta et al., 2017). In Colombian ‘barrios’, this resonates with findings where the involvement of fathers is limited (Concha‐Eastman & Concha, 2014).

### 1.2 The study

The paper reports on a study that looked at maternal health and family dynamics within a community context. It aims to show how a family systems approach adds to the evidence‐base regarding the role grandmothers play in maternal and infant feeding practices, as well as how they facilitate support systems beyond nutrition, enhancing maternal mental health. The study took place in the Eastern Periphery of Cali, Colombia. Socio‐economic conditions in this area differ from those of the city. More than 80% of its population is in the lowest social stratum, 1–2 [Departamento Municipal de Planeación (DMP), 2016]. Poverty, school exclusion and youth homicides are disproportionately higher compared with the city (Observatorio de Seguridad, 2019). Nevertheless, health provision has changed significantly in recent decades through the Social Security Health System, which provides coverage through contributory and subsidised programmes. About 40% of the area's population obtains subsidised healthcare (Secretaría de Salud de Cali, 2020). Healthcare access has contributed to maternal mortality reductions in Cali in recent years, although 10 out of 15 maternal deaths occurred in the area (Secretaría de Salud de Cali, 2020). Cali's Eastern Periphery presents a case to study how families function beyond the heteronormative model and how they adapt to health provision.

## 2 METHODS

### 2.1 Design and participants

The study applied a qualitative longitudinal design with a prenatal (T1) and postnatal (T2) timescale. T1 gathered the pregnancy experience, and T2 was carried out a year later to examine the postpartum period and infant caregiving. Individual in‐depth interviews were conducted with adolescent mothers and mothers in their 20s (*n* = 35 at T1; *n* = 21 at T2) and mothers' mothers [maternal grandmothers of the infant, locally known as ‘mamitas’ (i.e., mummies)] (*n* = 15 at T1; *n* = 12 at T2). All were the mothers of the pregnant women/mothers except for one older sister, a grandmother and an aunt. The profile of pregnant women and grandmothers mapped local demographics and is described in Table 1 below. Interviews were also conducted with peer community mothers, traditional birth attendants and community psychologists (*n* = 11), as well as medical and health promotion staff from the local hospital (*n* = 6), totalling 17 stakeholder interviews. Data collection was supported by fieldwork reflexive diaries. Interview guides were used for consistency across the board. The T1 schedule covered questions on demographics, the pregnancy (including diet and nutrition), childbirth expectations, family dynamics, community knowledge and health provision. The T2 schedule revisited T1 questions and added childbirth, postpartum experience, infant caregiving and feeding practices.

**TABLE 1:** Sample characteristics of mothers (*N* = 35) and grandmothers (GMs) (*N* = 15)

	Demographics	Number of participants
Mothers' sample (*N* = 35)		
Mother's age	16–20	*N* = 22
	21–24	*N* = 10
	25–29	*N* = 3
Mother's gestation at T1	Second trimester	*N* = 3
	Third trimester	*N* = 32
Mother's ethnic background	Afro‐Colombian	*N* = 15
	Mixed indigenous	*N* = 12
	Afro‐Colombian and mixed indigenous	*N* = 8
Mother's education	Some secondary school	*N* = 17
	Secondary school diploma	*N* = 6
	Further education/technical course	*N* = 12
Child's father involvement	Single/limited/no involvement	*N* = 19
	Some support	*N* = 6
	In a relationship with father	*N* = 10
Grandmothers' sample (*N* = 15)		
GM's age	35–39	*N* = 4
	40–44	*N* = 7
	45–49	*N* = 1
	50–54	*N* = 2
	55+	*N* = 1
GM's ethnic background	Afro‐Colombian	*N* = 7
	Mixed indigenous	*N* = 8
	Afro‐Colombian and mixed indigenous	*N* = 0
GM's education	Some secondary school	*N* = 10
	Secondary school diploma	*N* = 5

### 2.2 Data collection

Access to participants was facilitated by Cali's local health institution, the Eastern ESE (‘ESE Oriente’). Recruitment criteria included primiparous women, 16 years or over, with a subsample of 15 grandmothers. Participants were invited to participate in the research after antenatal appointments. All data were collected by the first author. Interviews were conducted in Spanish in health facilities, during a 2‐month period/phase at a convenient time for participants. Interviews lasted 25–70 min. Participants were offered a small gift as a gratuity. A pilot was conducted with five interviews at T1. Think aloud protocols (Ericsson & Simon, 1994; Fox et al., 2011) were applied in the post interview pilot. Support from health staff aided recruitment at T2, through phone calls reinviting women to participate. T2 was conducted a year after T1, when 21 of the 35 mothers were retained (60%) and 12 out of the 15 grandmothers (80%). An exercise of communicative validation (Kvale, 1995) at the health institution was carried out in T2. Research findings were well received and health staff pointed out the need to continue increasing exclusive breastfeeding in the maternal local population.

In qualitative research, reflexivity and the researcher's subjectivity are considered a resource rather than a limitation (Clarke & Braun, 2018; Shacklock & Smyth, 1998). Acknowledging the researcher's positioning (i.e., nationality, class, and gender) is important as it frames worldviews, interview dynamics and interpretation (Murray & Poland, 2006). Throughout the fieldwork, the first author positioned herself as a psychology researcher with a maternal health interest and as a Colombian mother. The authors' experience of working with similar communities and the first author's sharing participants' nationality facilitated communication, rapport and trust. It ensured the sensitivity and appropriateness of data instruments (Kvale & Brinkmann, 2009), which were reviewed with local stakeholders.

### 2.3 Data analysis

Verbatim transcripts were analysed in NVivo12. Thematic analysis followed Braun and Clarke (2006, 2014) by mapping a systematic coding framework, identifying patterns of meaning across the corpus. This was combined with Fereday and Muir‐Cochrane's (2006) hybrid analytical approach of deductive and inductive reasoning, enabling codification that is data and theory‐driven. Transcripts were reread, checked for ‘inaudibles’ and inaccuracies before uploading into the software. Analysis was based on the salience, distinctiveness and meaning of themes, identifying common and divergent voices, as well as maintaining relevance connecting with research objectives. Coding frame drafts were close to verbatim extracts, with numerous subcodes. As coding developed, the coding frame was revised, and text‐units were recoded to improve coherence. Main themes were retrieved to explore nuances and connections. To integrate T2 data, the coding frame was restructured to align with T1 codes to enable comparisons in the pre‐post design. Analysis was mainly conducted by the first author and jointly revised with the second author. For this paper, relevant coding was extracted and organised to fit the study's objectives. Final quotes were translated and back‐translated by the first author and reviewed by a bilingual colleague.

### 2.4 Ethical considerations

The research was approved by the Department of Psychological and Behavioural Science's ethics review board at the London School of Economics (LSE) as part of a doctoral degree, which was awarded to the first author on January 2019. It was also approved by the local health research ethics team at ESE Oriente in Cali, Colombia. The study was described to participants at recruitment and prior to obtaining consent. Confidentiality, privacy and anonymity were emphasised, as well as the right to withdraw at any point. All participants read and signed consent forms. Pseudonyms were chosen by the women to ensure anonymity.

## 3 RESULTS

The analysis yield four themes: (T1) maternal diets in pregnancy; (T1 & T2) grandmothers as decision‐makers in breastfeeding and infant feeding practices; (T1 & T2) grandmothers' scaffolding and (T1 & T2) how poverty impacts maternal diets and infant feeding. They are described below.

### 3.1 Maternal diets in pregnancy (T1)

Pregnant women recognised the importance of a healthy diet during pregnancy, attending antenatal appointments and reducing unhealthy practices. All pregnancies were unexpected except for three; participants reported feeling guilty upon receiving the news, as many had drunk alcohol and a few had smoked or taken psychoactive substances. Most declared they had suspended these practices, except for two who drank sporadically. The rest highlighted they found the smell of smoke and alcohol uncomfortable. Morning sickness impacted their diets and meant they avoided protein‐based foods or vegetables. During late pregnancy, participants in the 16–20 age range described sugar‐based cravings and ate packaged snacks from local corner shops. Some were concerned about weight management.

You get a lot of cravings and I love ice creams with too much cream, chocolate. […] The doctor told me that I was gaining more weight than expected and I needed a better diet. When I told my mum she reminded me of my dad's diabetes […] and for my baby I have been taking care of myself and eat better now, I buy fruits (T1, Ana, pregnant woman, 16–20 years).

Ana explained that she was receptive of health advice, given her genetic history. Ana indicated that her father left home when she was young and knowledge of her father came from her mother. The compounded health advice motivated Ana to change her diet by assuming the responsibility for her unborn child's health. This was common for more than half in the dataset.

About a third of the pregnant women pointed out they ate healthy foods they enjoyed. Those who stopped feeling morning sickness incorporated pulses into their diets. Vegetables, however, were disliked by many women.

I know I have to eat better but I don't like vegetables, only fruit, like pineapple. I like beans now, I couldn't [eat them] before but now I love beans and lentils! (T1, Yani, pregnant woman, 16–20 years).

Apart from food preferences, another maternal barrier to eating healthier was food preparation. To support healthier diets, grandmothers cooked, prepared healthier dishes or reinforced nutritional advice.

I make my salads and I always leave her [her daughter] some and I say to her, ‘I left you a salad, eat it, you know it's good for you’ (T1, Maria C, grandmother, 35–39 years).

She [her daughter] doesn't combine her foods well and has been underweight. I'm there on top of her, ‘remember that the nutritionist told you this and this,’ so that she eats well and now, thank God, she's put on a bit of weight (T1, Jenny, grandmother, 40–44 years).

Maria C and Jenny influenced their daughters to eat healthier food. This shows how grandmothers play a central role in maternal diets by asserting their family positioning.

### 3.2 Grandmothers as decision makers in breastfeeding and infant feeding practices

Regarding breastfeeding, 11 out of the 21 mothers interviewed at T2 exclusively breastfed for at least 5 months and six combined breastfeeding (from 3 to 5 months) with *bienestarina*, a local macronutrient supplement (Rozo, 2000). The main reason provided by the six mothers who combined breastfeeding with *bienestarina* was economic; they had to work. Access to and use of breast pumps is not common in the community. Four started bottle feeding with infant formula by the first month (one mother developed mastitis, another found it very painful and two highlighted infant rejection). All were complementary feeding (infants were 9–12 months old) in differing degrees.

She prepares the compotes, with fruits […]. We started giving her porridge, but it was too heavy, doctors told her best to wait a bit (T2, Nancy, grandmother, 40–44 years).

Although exclusive breastfeeding was not found across the sample, many depicted following biomedical advice and grandmothers supported breastfeeding practices. This included breastfeeding expectations and uptake, where some strongly influenced maternal breastfeeding expectations by T1.

I say to her [daughter]: ‘as long as you live here in my house with me, you are going to breastfeed the baby. Once you hold your baby in your hands and start breastfeeding, what you really feel when you are breastfeeding, is a wonderful thing, you'll see’ (T1, Stella, grandmother, 40–44 years).

Most grandmothers like Stella were key decision makers as heads of households, particularly with adolescent mothers, although a few maintained a more advisory approach. Here, however, Stella alluded to the maternal attachment enabled by breastfeeding, using her experience to encourage her daughter Karen. At T2, Karen reported Stella was central in her uptake of exclusive breastfeeding.

As for the care, I'm doing whatever my mum says, only breastmilk. Before I didn't want to but now I am glad I followed her (T2, Karen, mother, 16–20 years).

### 3.3 Grandmothers' scaffolding

So far, findings have illustrated the role grandmothers play given their powerful positioning impacting pregnancy diets and breastfeeding. However, this needs to be understood at a deeper level. Mothers to a great extent followed grandmothers' decisions not just due to the grandmothers' standing, but because they enable an environment of holistic support.

I see everything that's good in my mum. My mum, the guardian angel, she always gives everything for me, she has been the one who helps me a lot with my child (T2, Marlene, mother, 16–20 years).

In spite of the fact that one says, ‘oh, she [grandmother] can be tiresome with her nagging,’ but my mother's support has been essential to my baby's upbringing (T2, Grettel, 16–20 years).

The quotes represent a common maternal perspective, ranging from providing holistic support to essential help in caregiving, despite intergenerational tensions. In addition to infant caregiving, grandmothers provide maternal mental health support and encouragement.

My mother's support has been essential to my baby's upbringing. When I am wrong about something, she says, ‘Caro, this is not this way’ [….] When she is sick I start crying, it hurts me when she is sick but she tells me not to cry, look, you are stressing out ‘la niña’ [baby girl], she needs you, that encourages me (T2, Carolina, mother, 21–24 years).

Carolina expressed her emotional reaction when her mother is ill, reflecting how essential she is to the dyad. The grandmothers' support system is described as *scaffolding* mother and infant, from financial provision, knowledge and practices, to maternal psychological support.

#### 3.3.1 Intergenerational cultural practices

Grandmothers' scaffolding was prevalent across the sample. Data show how this was evident in maternal diets, nutrition advice, breastfeeding, caregiving and maternal psychological support. In this section, the supportive environment extends to postpartum cultural practices, which are an intrinsic part of intergenerational responsibility. The knowledge base of these practices relates to hot–cold polarities after childbirth (Callister, 2006; Callister et al., 2011; Cosminsky, 1976; Forero et al., 2018; Naser et al., 2012; Piperata, 2008). In childbirth, women lose blood, which is considered the body's warm fuel. Postpartum practices are carried out to restore their hot–cold bodily balance. In Colombia, this is known as ‘la dieta’ (the diet), a restrictive period based on homemade diets, maternal resting and infant caregiving. Below, a grandmother described the intergenerational knowledge chain of the practice.

My grandmother took care of the diet to get rid of ‘frios’ (the colds). She gave us herbal drinks with lots of cinnamon, which help one close one's bones [after delivery], celery and parsley water to expel all that really bad blood and truth be told one drinks that ‘agüita’ (water) and you get rid of all that and feel anew [...] And so with my nieces and my daughter I give them during the early days parsley water (T2, Rosa, grandmother, 45–49 years).

Rosa described a meaningful practice representing a protected time in a confined space for physical and psychological recovery from childbirth. Although herbal waters are not a nutritional staple, they serve as caregiving tools to facilitate maternal transitions. All grandmothers enacted these intergenerational practices, though a few indicated their daughters were not receptive of some rituals, regarding these as old fashioned and outdated.

### 3.4 How poverty impacts maternal diets and infant feeding

On a material level, a new child can raise concerns about the family's future. Most grandmothers were worried about an imminent financial squeeze, given unstable low household incomes.

I wanted something different for her, not to get pregnant so quickly. I get stressed, the rent, bills arrive and my work is not stable, so today ok, tomorrow no, there are weeks that we eat well, there are weeks that we don't, that stresses me because now we will have another mouth to feed (T1, Fabiola, grandmother, 40–44 years).

Fabiola's extract shows how most households depend on single women on low incomes based on the informal economy, where news of another child adds to financial stress.

Economic difficulties were the norm across the sample. Some health practitioners have this contextual awareness and indicated that they tailor advice by describing the possibilities of accommodating a healthier diet to low incomes.

I give them [pregnant women] recipes, grab an avocado, grab some berries, milk worth 1,000 [30 US cents] and blend all that. ‘Ah, cool, it tastes delicious!’ [They say]. ‘And it will nourish you more, how much is the Coca‐Cola and donut worth, the *Tampico* [branded juice]? Much more than 1,000′. ‘And boiled eggs? An egg is 200, you can cook eggs or peel the avocado, do it’ (T1, Female Nutritionist, Health System).

The nutritionist depicts how she grounded her advice in financial incentivising terms so it resonated more with women. She aimed to counteract the representation that all healthy foods are expensive. Tropical fruits in an equatorial country like Colombia are abundant and can be purchased at lower prices in street markets.

Yet a few discussed recurrent health conditions related to unaffordability of prescriptions, formula milk (for those not breastfeeding), or given infrequent nappy changing, a fungal infection. Other health issues related to iron deficiencies.

About 20 days ago he [child was ten months old] got sick and in the tests they said he had low haemoglobin, so they asked me to give him things that have iron, pulses, meat if I could […] But meat is too expensive (T2, Patricia, mother, 21–24 years).

Patricia's point about the unaffordability of meat underlines yet again how poverty impacts feeding practices in relation to nutritional advice. Though pulses are mentioned, meat is preferred.

#### 3.4.1 Adapting infant feeding in context

Unaffordability signals the need for increasing household incomes, expressed by eight grandmothers. They thought it was time for mothers to contribute economically to the household. For this to occur, some of them implement a strategy to reduce breastfeeding and increase complementary feeding.

I say to her, ‘you have to feed him [more solids], because he has only breastfed and only breastfed’, now he eats savoury foods that one gives him, but not much […]. I give him *calditos* (broth‐based soups) that I make […]. I told her ‘I'm giving him more foods because he needs it and so you can start looking for a job’ because it's already very difficult to keep them both (T2, Fabiola, grandmother, 40–44 years).

Fabiola again highlights financial constraints and calls on her daughter to take on a job. Others take a route which does not directly alter infant feeding, but by maintaining their positioning, they insist on mothers returning to employment, impacting complementary feeding.

She has given him her milk like I gave them my milk. But now she has to return to work, she knows she has to return, I insist (T2, Sandra, grandmother, 35–39 years).

Doctors tell me that I should breastfeed till he is two years old, but I need to work (T2, Katerine, mother, 21–24 years).

Although breastfeeding (exclusive/continued) has been welcomed, grandmothers indicate they cannot maintain daughters as stay‐at‐home mothers and some mothers like Katerine agree. Yet, for those who disagree, the grandmothers' scaffolding also involves setting limits.

## 4 DISCUSSION

The study explored the role grandmothers play in maternal nutrition and infant feeding practices, as well as the other support systems they provide influencing MCN and maternal mental health. First, it introduced maternal diets and revealed how grandmothers were enablers of a supportive maternal–infant environment. Next, it identified the impact of poverty on feeding practices. Specifically, it highlighted how grandmothers are also pragmatists, by adapting infant feeding practices to meet the family's economic needs.

Findings resonate with research suggesting that grandmothers play a central role in nutrition for many families in the Global South during the prepartum, postpartum, breastfeeding or complementary feeding periods (Aubel, 2012; Aubel et al., 2004; Bezner Kerr et al., 2008; Chakona, 2020; Faye et al., 2019; Gupta et al., 2015; Ijumba et al., 2014; Karmacharya et al., 2017; MacDonald et al., 2019; Martin et al., 2015). In relation to maternal diets, research has shown that dietary habits in adolescents reflect their preference for ‘modern’ foods and snacking, given widespread availability with globalisation and markets (Blum et al., 2019). This issue has been identified in the nutrition transition literature, which shows how global development has shifted human diets towards unhealthy consumption patterns (i.e., fats, sweeteners and processed foods) in the Global South (Hawkes, 2006; Popkin, 1993, 2006). Against this backdrop, the research identifies the role that nutritionists—reinforced by grandmothers—play in improving maternal diets.

The research connects with studies in Colombia (Cháves, 2019; Forero et al., 2018) and other countries (Aubel, 2012; Aubel et al., 2004) indicating that grandmothers are largely involved with feeding practices and caregiving. Aligned with such practices is their support to the dyad across various dimensions, which the study identifies as grandmothers' scaffolding, a concept that has not been used in this manner in MCN research. This derives from psychosocial scaffoldings (Jovchelovitch & Priego‐Hernández, 2013), which sharpen our understanding of the practical, psychological and cultural support system found. Psychosocial scaffoldings show how grandmothers provide encouragement and caregiving, set limits and maintain cultural practices, such as the diet, which support maternal transitions. Diet practices have been culturally maintained across Global South regions (Callister, 2006; Callister et al., 2011; Cosminsky, 1976; Forero et al., 2018; Naser et al., 2012; Piperata, 2008) providing a framework for protection, guidance and support for new mothers and infants. Diet practices have, however, also been impacted by global forces, as the study found in relation to snacking (Hawkes, 2006; Popkin, 1993, 2006). Engaging with psychological factors at the symbolic, cultural and material level is therefore fundamental to analysing the currency of family–community support systems in the current global context.

The study adds to the evidence‐base supporting the transferability of a family systems approach in the Global South (Aubel, 2012; Aubel et al., 2004; Karmacharya et al., 2017; MacDonald et al., 2019) through conceptual frameworks that sharpen the rationale for grandmother inclusion in MCN programmes. This is relevant in Colombian and other South American peripheries because many fathers are rarely involved in child provision (Chant & Craske, 2003; Chant & McIlwaine, 2015; Cuesta et al., 2017), and grandmothers take on *the third role* in supporting the dyad. The third role refers to grandmothers who occupy a family positioning supporting the first two roles (infant and mother). Roles help people define who they are in relation to others in a given group or category (Hogg, 2003; Reicher et al., 2010; Tajfel, 2010; Tajfel & Turner, 1986). The grandmother's role represents an essential family positioning in MCN. In Colombia, however, grandmother initiatives are still scarce (Cháves, 2019). In Cali, programmes continue to target the mother–child dyad to the exclusion of grandmothers and the family (Advisor to Health Secretariat, personal communication, 2020).

Findings illustrate the value awarded to cultural practices but also highlight how grandmothers are receptive to biomedical advice. A few Colombian studies on MCN have shown how families use both biomedical and community knowledges, highlighting that when the former contradicts grandmothers', community knowledges prevail (Becerra‐Bulla et al., 2015; Forero et al., 2018). Other studies have found that when both knowledges are aligned, better nutritional outcomes are possible (Karmacharya et al., 2017). This resonates with the wider CHP perspective suggesting we need to move away from binary notions of knowledges and practices for better application of health behavioural programmes (Campbell & Cornish, 2014). Biomedical and community knowledges are not mutually exclusive because families accommodate to multiple knowledges as they fit different purposes (Jovchelovitch, 2007). One addresses physical health, the other identities and wider health such as psychological and social wellbeing.

Breastfeeding is critical for child health and development (Victora et al., 2016; WHO et al., 2018) and after exclusive breastfeeding, continued breastfeeding is recommended up to 2 years (Rollins et al., 2016; WHO et al., 2018). Nonetheless, findings show this is challenging when economic stressors affect low‐income families. The study presents grandmothers' pragmatic response to accommodate feeding practices with economic needs. Balancing the implementation of optimal health advice in impoverished settings has represented a conundrum for the nutrition field (Bhutta et al., 2013). Perhaps by embracing local ‘messy’ complexities (Nolas, 2014), given interrelated factors such as low‐income households, maternal youth unemployment and optimal feeding practices (Agunbiade & Ogunleye, 2012; Amjad et al., 2019; Brauner‐Otto et al., 2019; Forero et al., 2018), this may be possible. It would mean applying a more practical approach (Cornish, 2009) to the best possible fit of health guidelines. This would include direct responses to poverty and adversity stressors (Lund et al., 2018). Policy makers could thus tailor interventions by committing to flexibility in adopting programmes that can work for people in practice.

Finally, a global review found that nutrition programmes should ideally be scaled up to address both nutrition and mental health (Bhutta et al., 2013). By applying a family systems approach, this study rendered visible the scaffolding grandmothers provide, including psychological support; this resonates with larger maternal mental health programmes (Hassan et al., 2016; Lund et al., 2018; Rahman et al., 2008), the inclusion of sociocultural domains as part of the social determinants of mental health (Lund et al., 2018) and caregiving in early childhood development (WHO et al., 2018). Given the challenges faced by families in caring for infants and children in impoverished settings in the Global South (WHO et al., 2018), this paper calls for MCN programmes to continue their focus to support *the caregivers' carers*, who can provide additional routes for maternal mental health. The paper also touches upon a wider issue. The failure to recognise grandmothers' scaffolding disregards the third role they uphold for MCN in families experiencing economic constraints. We know recognition of grandmothers' scaffolding can better inform the ways in which health inequalities in the long term are addressed. We simply need to continue our efforts to obtain that recognition.

## CONFLICTS OF INTEREST

The authors have no conflicts of interests to report.

## CONTRIBUTIONS

NC wrote the manuscript, conceived and designed the research and performed the data analysis. SJ supervised the research, contributed to the conceptual framework and provided critical feedback. NC and SJ revised and approved the final version of the manuscript.

## Data Availability

Data available on request due to privacy/ethical restrictions: the data that support the findings of this study are available upon reasonable request from the corresponding author. The data are not publicly available due to privacy and ethical restrictions compromising the confidentiality and anonymity that was guaranteed to participants during data collection.
